# Intracyclic Velocity Variation and Arm Coordination for Different Skilled Swimmers in the Front Crawl

**DOI:** 10.2478/hukin-2014-0111

**Published:** 2014-12-30

**Authors:** Yuji Matsuda, Yosuke Yamada, Yasushi Ikuta, Teruo Nomura, Shingo Oda

**Affiliations:** 1 Department of Sports Sciences, Japan Institute of Sports Sciences, Tokyo, Japan.; 2 Japan Society for the Promotion of Science, Tokyo, Japan.; 3 Graduate School of Education, Osaka Kyoiku University, Osaka, Japan.; 4 Graduate School of Science and Technology, Kyoto Institute of Technology, Kyoto, Japan.; 5 Faculty of Health and Well-being, Kansai University, Osaka, Japan.

**Keywords:** intracyclic velocity variation, arm coordination, swimming, performance

## Abstract

The aim of this study was to examine whether the intracyclic velocity variation (IVV) was lower in elite swimmers than in beginner swimmers at various velocities, and whether differences may be related to arm coordination. Seven elite and nine beginner male swimmers swam front crawl at four different swimming velocities (maximal velocity, 75%, 85%, and 95% of maximal swimming velocity). The index of arm coordination (IDC) was calculated as the lag time between the propulsive phases of each arm. IVV was determined from the coefficient of variation of horizontal velocity within one stroke cycle. IVV for elite swimmers was significantly lower (26%) than that for beginner swimmers at all swimming velocities (p<0.01, 7.28 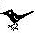
1.25% vs. 9.80 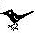
1.70%, respectively). In contrast, the IDC was similar between elite and beginner swimmers. These data suggest that IVV is a strong predictor of the skill level for front crawl, and that elite swimmers have techniques to decrease IVV. However, the IDC does not contribute to IVV differences between elite and beginner swimmers.

## Introduction

Swimming velocity is not stable, but fluctuates, even in a stroke cycle. A larger intracyclic velocity variation (IVV) may be disadvantageous as velocity fluctuation leads to increased inertia and drag (Nigg, 1983) and higher energy costs (Barbosa et al., 2005; Vilas-Boas, 1996). However, there are controversies over the relationship between IVV and swimming performance. For example, Takagi et al. (2004) reported that faster swimmers showed lower IVV in breaststroke, while Lebalanc et al. (2007) reported that elite swimmers showed a higher maximal peak velocity in a stroke cycle, resulting in higher IVV in breaststroke. Thus, these studies suggest that IVV for elite swimmers may or may not be lower than beginner swimmers, despite the fact that swimming with low IVV is related to lower swimming efficiency, defined as lower energy cost and drag at a given swimming velocity.

There are few studies examining the relationship between IVV and swimming performance in the front crawl. Holmer (1979) compared two swimmers and reported that IVV was lower for the faster swimmer than that the slower one at velocities of 1.2 and 1.6 m/s. However, only two swimmers were compared in that study, with no statistics. Schnitzler et al. (2010) reported that IVV of elite swimmers was significantly lower than that of recreational swimmers. On the other hand, Psycharakis et al. (2009) examined the relationship between IVV and swimming velocity for a 200 m race, and reported that IVV was not correlated with swimming velocity. However, the authors stated that the subjects in their study were too homogenous, and further research was required in swimmers of different skill levels. As such, IVV should be compared between two groups with different skill levels and a sufficient number of subjects. In addition, IVV was compared at just one submaximal velocity in the study by Psycharakis et al. (2009). There are also data suggesting that IVV for elite swimmers did not change with increasing swimming velocity (Schilezler et al., 2008), although it remains unclear whether such a response in IVV might be observed in beginner swimmers. Thus, the difference of IVV between different swimmer skill levels at various velocities, which includes maximal velocity, remains unknown.

Arm coordination may influence IVV because it has been demonstrated to influence performance of front crawl (Chollet et al., 2000; Miller et al., 2002; Seifert et al., 2007). The nonpropulsive phase of highest performers has been shown to be shorter than that of lowest performers at various velocities (Chollet et al., 2000), which might lead to a decrease in velocity in a stroke cycle and an increase in IVV. Arm coordination is quantified using an index of coordination (IDC), defined as the lag time between propulsive actions of the right and left upper limbs (Chollet et al., 2000). The difference in IVV for different skill levels in swimming may be associated with the IDC.

The aims of the present study were to examine the difference of IVV between elite and beginner swimmers at various swimming velocities, including maximal velocity, and to examine the contribution of the IDC to the variability of IVV.

## Material and Methods

Seven elite swimmers (age: 20.9 ± 0.9 years, body height: 1.75 ± 0.05 m, and body mass: 67.9 ± 6.0 kg) and nine beginner swimmers (age: 20.2 ± 1.6 years, body height: 1.72 ± 0.04 m, and body mass: 63.9 ± 3.5 kg) were voluntarily enrolled in this study. The best performance of the elite swimmers and beginner swimmers for the 100 m crawl was 54.5 ± 1.3 s and 64.5 ± 3.5 s, respectively, corresponding to 86.3% and 72.9% of the world record. The protocol was explained in full to all swimmers before testing and they provided written consent to participate in the study. This study was performed in accordance with the ethical standards and approved by the local Ethics Committee of Graduate School of Human and Environmental Studies, Kyoto University.

### Swim test

The subjects swam 30 m front crawl at four velocities in a 50 m pool beginning from a push of the wall. The four velocities used were maximal velocity (Vmax) and 75%, 85%, and 95% Vmax (V1, V2, and V3, respectively). This 30 m was divided into three phases, as follows: a start phase, from 0–10 m; a swimming phase, from 10–25 m; and a finish phase, from 25–30 m. First, all subjects swam a 30 m length front crawl at Vmax. All subjects then swam three additional 30 m trials at V1, V2, and V3. The subjects were asked to attain each target time and constant velocity from 10–25 m. If the performed time was not within the range of time corresponding to ± 3% of the target velocity (e.g., from 82–88% of target velocity at V2), the trial was repeated until their swimming velocity fell inside the range. The swimmers were asked to hold their breath for the swimming phase to avoid modifications in arm coordination due to breathing (Chollet et al., 2000; Seifert et al., 2004, 2007). The trials were started in the water, without diving.

### Video analysis

Two underwater video cameras (Yamaha, Shizuoka, Japan) collecting at 60 Hz were used to film the swimmers from the right side and left side views. Both cameras were allowed to pan, and were connected to a video timer, a video recorder, and a monitoring screen. A large test section (8.0 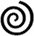 1.0 m2) between 15–23 m of the 30 m was defined to cover two complete stroke cycles. Only one camera, which filmed the swimmers from the right side, was used for digitizing, while both cameras were used to analyze classification of the arm movements.

A panning periscope system based on Yanai et al. (1996) was used for data collection. A control object (2.0 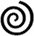 1.0 m2) with 30 control points was placed in four consecutive locations along the 8.0 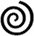 1.0 m2 calibration space, and was recorded while the camera was panning. Eight reference markers were placed at 1.0 m intervals along the calibration space. The reference markers were used to define the origin and the horizontal axis of a global reference system. Two-dimensional coordinates were obtained by digitizing 30 control points for each field and each consecutive location (Yanai et al., 1996; Yu et al., 1993). The images were manually digitized at 60 Hz by a single researcher (Frame-Dias II software; DKH, Tokyo, Japan) for the two stroke cycles. The subjects wore a swimming cap with a marker placed on the head approximately 5 cm above the right ear for all trials. For the videotapes of the performances, both the marker placed on the swimmers head and the reference markers were digitized for each field. The marker attached to the swimming cap remained under water and was visible all of the time. A low pass Butterworth filter with a cut-off frequency of 8 Hz was used for analysis of the horizontal velocity of the head, as previously reported (Berger et al., 1997).

IVV was quantified by determining the coefficient of variation of the horizontal velocity (Schnitzler et al., 2010). The average swimming velocity in a stroke cycle (SV) and the stroke rate (SR) were measured over two stroke cycles, and stroke length (SL) was calculated as SV/SR.

### Coordination of arm movements

Arm coordination was quantified using the IDC, as defined by Chollet et al. (2000). The beginning of the propulsive phase was defined as when the hand began the backward movement. The end of the propulsive phase was defined as when the hand released from the water. Duration of a complete stroke cycle was calculated as the mean of duration from the timing of the first right hand entry into the water to the second right hand entry into the water, and from the timing of the first left hand entry into the water to the second left hand entry into the water.

The IDC was calculated as the mean of the time between the beginning of propulsion in the first right arm stroke and the end of propulsion in the first left arm stroke (IDCleft;; equation 1), and the time between the beginning of propulsion in the second left arm stroke and the end of propulsion in the first right arm stroke (IDCright; equation 2). The IDC was expressed as percentage of the mean duration of the strokes.
[1]
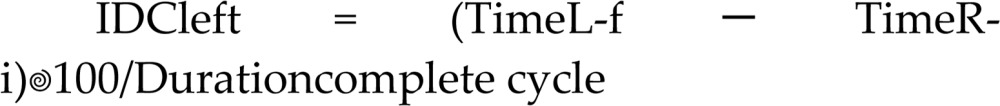

[2]
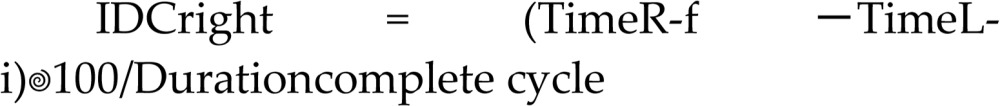


where L and R refer to left and right arm, i and f refer to the beginning and ending of the propulsive phase.

When there was a lag time between propulsive phases of the two arms, the stroke coordination was termed ‘catch-up’ (IDC < 0). When the propulsive phase of one arm started at the time the other arm finished its propulsive phase, the coordination was termed ‘opposition’ (IDC = 0). When the propulsive phase of the two arms overlapped, the coordination was termed ‘superposition’ (IDC > 0) (Chollet et al., 2000).

### Statistical analysis

Means and standard deviation were calculated for all the measured and calculated variables. The differences between the two groups and between the four velocities for SV, SR, SL, IVV, and IDC were examined by a mixed model two-way ANOVA. Velocity conditions (V1, V2, V3, and Vmax) were treated as within-subjects variables, and the performance level (Elite, Beginner) was treated as a between subjects variable. When the two-way ANOVA revealed a significant interaction, groups were evaluated separately and Tukey’s post-hoc used to examine differences between velocity conditions. Trend analyses for linear were also conducted. For all analyses, p<0.05 was used to indicate statistical significance. All analyses were performed using SPSS 12.0 (SPSS, Chicago, IL, USA). The η2 (Eta squared) was also calculated as described by Cohen (1988), where a small effect size corresponds to an η2 = .0099, a medium effect size to an η2 = .0588, and a large effect size to an η2 =.1379. The threshold for significance was set at the 0.05 level of confidence.

## Results

### Velocity, stroke rate, and stroke length

**Figure 1:**
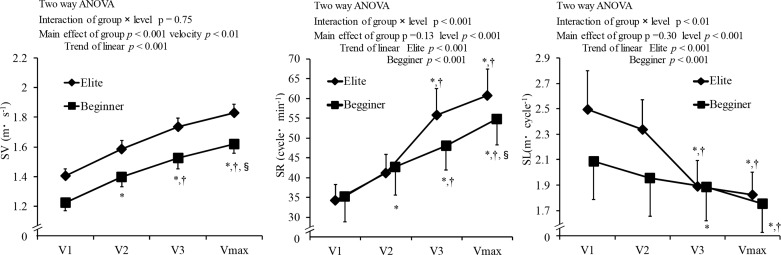
Average swimming velocity (SV), stroke rate (SR), and stroke length (SL) for elite and beginner swimmers at different swimming velocities. Vmax, maximal velocity; V1, V2 and V3, 75%, 85%, and 95% of the maximum velocity, respectively. * significant difference with V1; †, significant difference with V2; §, significant difference with V3 (p<0.05).

Average swimming velocity (SV), the stroke rate (SR), and stroke length (SL) are shown in Figure 1. The interaction between group and velocity was observed in the SR and SL (p<0.05). Although both elite and beginner swimmers increased the SR and decreased SL while increasing SV, the changing rate of the SR and SL for elite swimmers was significantly higher than for beginner swimmers. There was no difference in the SR at all velocities between elite and beginner swimmers. SL for elite swimmers at V1 and V2 was significantly higher than that for beginner swimmers. SV for elite swimmers was significantly higher than that for beginner swimmers for all velocities.

### IVV

**Figure 2:**
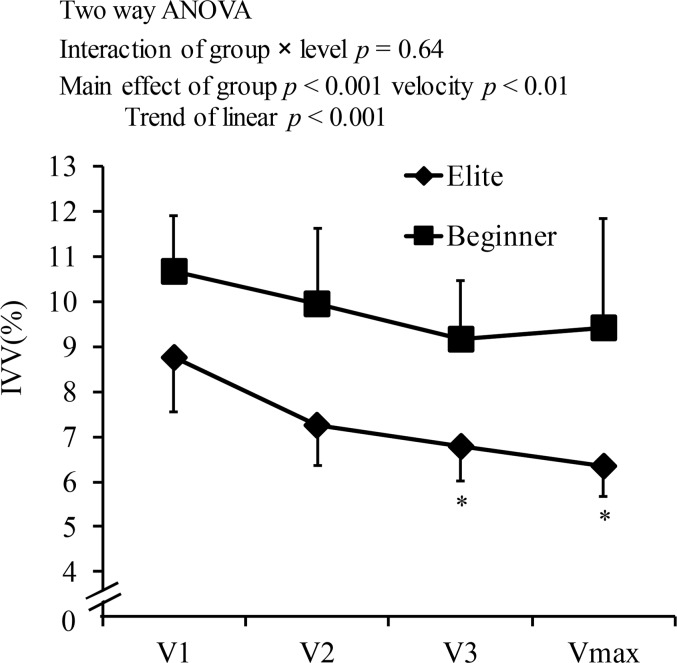
Intracyclic velocity variation (IVV) for elite and beginner swimmers at different swimming velocities; Vmax, maximal velocity; V1, V2 and V3, 75%, 85%, and 95% of the maximum velocity, respectively. * significant difference with V1 (p<0.05).

No interaction of IVV with group and velocity was observed (F(3,24) = 0.64, p = 0.593), and there was a significant main effect of group (F(1,14) = 29.22, p<0.001, η2 = 0.676), suggesting that IVV was smaller for elite swimmers than for beginner swimmers, and that the change in IVV with increasing SV did not differ with different swimming levels (Figure 2). The mean IVV at all velocities for elite swimmers was 26% lower than that for beginner swimmers (p<0.001, 7.28 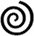 1.25% vs. 9.80 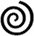 1.7%, respectively). The main effects of velocity for IVV were also significant (F(1,3) = 12.13, p < 0.01, η2 = 0.323). The Tukey’s post hoc test revealed that IVV at V3 and Vmax were significantly lower than that at V1. IVV for elite and beginner swimmers decreased 18% from 9.82% (V3) to 8.07% (Vmax) with increasing SV.

### IDC

**Figure 3:**
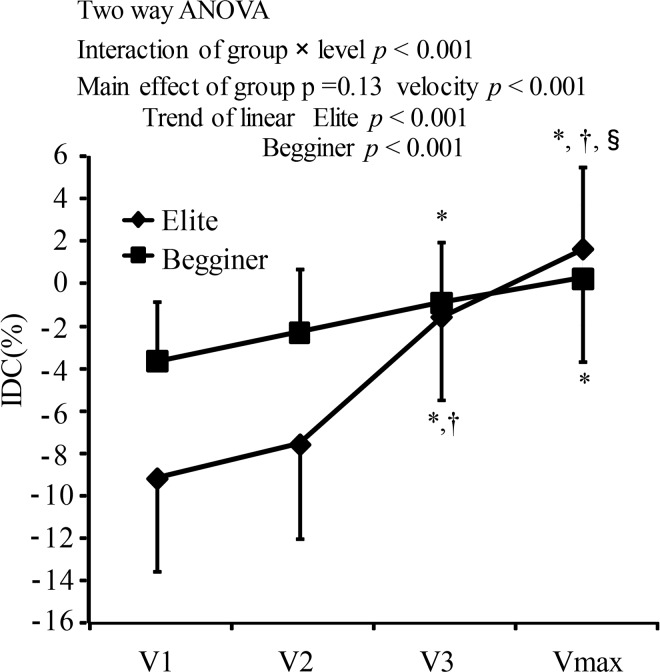
The index of coordination (IDC) for elite and beginner swimmers at different swimming velocities; Vmax, maximal velocity; V1, V2 and V3, 75%, 85%, and 95% of the maximum velocity, respectively. * significant difference with V1; †, significant difference with V2; §, significant difference with V3 (p<0.05).

A mixed model two-way ANOVA (group × velocity) showed significant interactions of the IDC with group and velocity (F(3,24) = 12.065, p<0.001, η2 = 0.165) (Figure. 3). Subsequent oneway ANOVA for each group showed a significant main effect of velocity for both elite swimmers and beginner swimmers (p<0.01). The post hoc test revealed that the IDC of elite swimmers at Vmax was positive and significantly higher than that at V1, V2, and V3, and that the IDC at V3 was significantly less negative than that at V1 and V2 (p<0.05). For beginner swimmers, IDC at V3 and Vmax was significantly less negative than that at V1 (p<0.05). These data suggest that the IDC changed from negative to less negative or positive for both elite and beginner swimmers, but the increase rate of the IDC was higher in elite swimmers than in beginner swimmers (IDC increased by 10.78% from −9.15% (V1) to 1.63% (Vmax) for elite swimmers; the IDC increased by 3.92% from −3.65% (V1) to 0.27% (Vmax) for beginner swimmers). There was no difference in the IDC between elite and beginner swimmers for each swimming velocity.

## Discussion

In the present study, IVV for elite swimmers both at maximal and submaximal swimming velocities was significantly lower than that for beginner swimmers (p<0.001, η2 = 0.676). These data suggest that lessening IVV is essential to achieve high swimming performance in front crawl. Lower IVV has several potential advantages. For example, lower IVV was reported to lead to decreased inertia and drag to be overcome by the swimmers (Nigg, 1983). Further, Fujishima and Miyashita (1999) simulated velocity transition calculated using estimated propulsive and drag force, and reported that less velocity fluctuation lead to higher average velocity. Barbosa et al. (2005) also reported that energy cost, as calculated by dividing total energy expenditure by velocity, was positively correlated with IVV, and suggested that higher IVV was related to low swimming economy.

Psycharakis et al. (2009) reported that IVV was not correlated with SV for the 200 m race of front crawl. By contrast, there was a difference in IVV between elite and beginner swimmers in the present study. The group of swimmers investigated by Psycharakis (2009) was homogeneous, and their swimming performance (125.4 ± 4.7 s for the 200 m race, corresponding to 81.3% of the world record) was intermediate when compared with the subjects in the present study (86.3% of the world record for elite swimmers; 72.9% of the world record for beginner swimmers). The authors stated that the subjects were too homogenous, and further research was required on swimmers of different skill levels. In the present study, when IVV was compared between swimmers at different levels, higher skilled swimmers exhibited lower IVV. Schnitzler et al. (2010) reported the elite swimmers had lower IVV than recreational swimmers. The present study supported their findings rather than the findings obtained by Psycharakis et al. (2009).

It was found that the IDC was not higher in elite swimmers than in beginner swimmers. The difference in IVV between elite and beginner swimmers was not related to arm coordination, suggesting that elite swimmers used alternative techniques to swim with lower IVV. Schnitzler et al. (2008) reported that although the IDC for females was more negative than that for males, the elite females showed a tendency toward lower IVV than the elite males at the same velocity (p = 0.06). Furthermore, in that study lower IVV for female swimmers was not related to the IDC. They suggested that lower IVV for females might be associated with lower drag. Cappert et al. (1996) reported that elite swimmers achieved a faster swimming velocity using better whole body streamlining to reduce the drag force from the water. Lower IVV for elite swimmers in the present study may be a result of lower drag force with better streamlining of body during swimming. However, we did not measure drag force and/or body form, and thus, further studies are required to determine to relationship between IVV and kinetic parameters.

It was previously shown that the IDC for elite swimmers was significantly less negative than that for beginner swimmers at various velocities (Chollet et al., 2000; Seifert et al., 2007), which is inconsistent with the results of the present study. These discrepancies may be explained by the stroke rate, as the IDC is more associated with high stroke rate values rather than with the skill level (Potdevin et al., 2006). As there was no difference in the stroke rate between elite and beginner swimmers at all velocities in the present study, there was a similar IDC between elite and beginner swimmers at all velocities.

For both elite and beginner swimmers, IVV at Vmax and V3 were significantly lower than that at V1, and did not change from V2 to Vmax. The Eta squared was 0.323, indicative of a medium effect size. Schnitzler et al. (2008) reported that both male and female elite swimmers maintained their IVV during increased swimming velocity in front crawl; the range of velocities (1.27–1.78 m/s) and the number of participants in that study was similar to that in the present study. Thus, it remains unknown whether IVV is decreased or maintained with increasing swimming velocity. Nevertheless, these data suggest that IVV does not increase with increasing SV in front crawl.

In the present study, the IDC for both elite and beginner swimmers changed from negative to positive, indicating that duration of the nonpropulsive phase in a stroke cycle decreased. Schnitzler et al. (2008) reported that IVV did not change, while the IDC increased, with increasing SV for elite swimmers. The authors suggested that if swimmers did not modify their arm coordination at high swimming velocity, the velocity in a stroke cycle would decrease markedly due to higher drag caused by increased velocity (Clarys, 1979), and IVV would increase. The effect of duration of the non-propulsive phase on IVV was not examined in the present study. However, IVV for the longer duration of the nonpropulsive phase at high swimming velocity is considered to be larger than that for shorter duration of the non-propulsive phase, since a longer non-propulsive phase indicates that the swimmer received prolonged drag resistance, without propulsive force. Modifying the IDC, indicative of a decrease in the duration of the nonpropulsive phase, would be related to the situation where IVV did not increase with increasing SV.

The IDC in elite swimmers increased more significantly than for beginner swimmers in the present study. Millet et al. (2002) reported that the increased rate of the IDC for elite swimmers was higher than that for triathletes around maximal velocity. These data suggest that the higher increase rate of the IDC was dependent on the skill level.

## Conclusion

IVV for elite swimmers was lower than that for beginner swimmers, while the coordination of arm movements did not differ between the two groups at all swimming velocities. These data suggest that lower IVV is required to achieve high swimming performance, and that differences of IVV are not related to arm coordination in front crawl. There was also no difference in the change in IVV with increasing SV between skill levels. By contrast, an increased rate of the IDC differed with the skill level, suggesting that the IDC was dependent on the skill level.
